# The Panama Pacific Dental Congress

**Published:** 1912-10-15

**Authors:** Frank L. Platt

**Affiliations:** San Francisco


### THE PANAMA PACIFIC DENTAL CONGRESS.

BY FRANK L. PLATT, D.D.S., SAN FRANCISCO.

In conversation a few days ago, with a gentleman from one of the Eastern States, he made the following remark: "Doctor, do you know there are hundreds of thousands of people living east of the Missouri River who, if only they knew of the opportunities out here and what a delightful place this is in which to live, would pack up and come out here and stay?"

We who are so fortunate as to stay here know so well the opportunities and delights of life on the Pacific Coast that the portion of his remark relating to them may pass unnoticed, but the words "if only they knew" are so pregnant with meaning, they open up such possibilities for publicity and exploitation, they suggest so much that may be done to promote the comfort and happiness of thousands of people, that they should not pass unheeded.

Each Pacific Coast State is an empire in itself with room for thousands of people and hundreds of enterprises to make comfortable and profitable homes; there are thousands of acres of mellow soil sleeping idly in the sun awaiting the hand of the farmer to awaken them to the activity of prolific harvests; there are mines and forests and opportunities for business enterprises of every sort needing only the attention of those who should be told of them to yield their portion of the wealth of the Coast and contribute their share to the enterprise and industry of humanity.

The Panama-Pacific Exposition will bring thousands of the people from "east of the Missouri River" to the Pacific Coast and many will stay here for their own good and the good of those already here, but that the greatest good may result from the advertising the exposition will give, it is essential that every feature of the exposition be made an unqualified success. It is seldom that professional men have an opportunity to take an active part in any great enterprise, particularly one of such far-reaching importance; their field of labor is naturally circumscribed and their occupation is such that they have little time to devote to interests outside of the daily routine of their professional activities, but in the Panama-Pacific Exposition an opportunity is presented every dentist on the Pacific Coast to do his part in helping to make the Panama-Pacific Dental

Congress the one feature of the exposition in which the profession is most vitally interested, an unqualified success, thus aiding in the success of the whole exposition. Every one who visits the exposition will find in its exhibits, amusements and congresses a vast amount of entertainment and information, but it is those who come for a special purpose, to study some particular exhibit or attend a congress devoted to a subject in which they are personally interested who will carry away the most lasting impression of the practical value of such an exposition.

The Panama-Pacific Dental Congress will be the greatest meeting of its kind ever held, and there are thousands of dentists who "if only they knew" what is being prepared for their information, amusement and general benefit by the Committee of Organization would make a special effort to attend the congress. Every dentist on the Pacific Coast should become a booster for this congress; he should spare no effoit to let others know what is being done or to induce them to come to San Francisco in 1915.

The Dental Gazette will give all the publicity it can to the exposition and the congress, but the entire profession of Pacific Coast should aid in calling the attention of those both in and out of the profession to what is being prepared for their entertainment and instruction.

Remember that "if only they knew" there are thousands who would come here, and see that they have the desired infoimation at every opportunity which presents itself.—*Pacific Dental Gazette.*

